# Molecular simulation study on CO_2_ sequestration and shale gas displacement in the quartz–calcite composite system

**DOI:** 10.1039/d6ra04585e

**Published:** 2026-07-13

**Authors:** Yifeng Ma, Caili Dai, Jianwei Gu

**Affiliations:** a Shandong Key Laboratory of Oil and Gas Field Chemistry, Department of Petroleum Engineering, China University of Petroleum (East China) Qingdao 266580 China daicl@upc.edu.cn gjwLcp@upc.edu.cn

## Abstract

Industrialization has raised atmospheric CO_2_ levels and worsened global warming, driving the urgent demand for CO_2_ emission reduction. CO_2_ enhanced shale gas recovery (CO_2_-ESGR) simultaneously boosts gas production and CO_2_ storage. This study constructs a quartz calcite composite nanopore model and employs GCMC, MD, and DFT simulations to investigate CO_2_ adsorption and shale gas displacement. The results show that CO_2_ adsorption follows the Langmuir isotherm. CO_2_ preferentially adsorbs on pore surfaces (especially calcite) with an asymmetric distribution. The isosteric heat for CO_2_ declines with the elevation of pressure and water content. When the moisture content is 15 wt%, compared to the dry state, the absorption of CO_2_ decreases by 45.9% because water can significantly inhibit adsorption. This indicates that compared to CO_2_ molecules, H_2_O molecules preferentially occupied the adsorption sites. DFT calculations indicate that the adsorption energy of CO_2_ on both quartz and calcite surfaces is lower than that of CH_4_ and C_2_H_6_. The electronic structure analysis reveals the microscopic essence of the preferential adsorption of CO_2_. It provides a theoretical basis for a deeper understanding of CO_2_ sequestration in shale reservoirs and the study of CO_2_-ESGR.

## Abstract 2

Industrialization has raised atmospheric CO_2_ levels and worsened global warming, driving the urgent demand for CO_2_ emission reduction.

## Graphical abstract

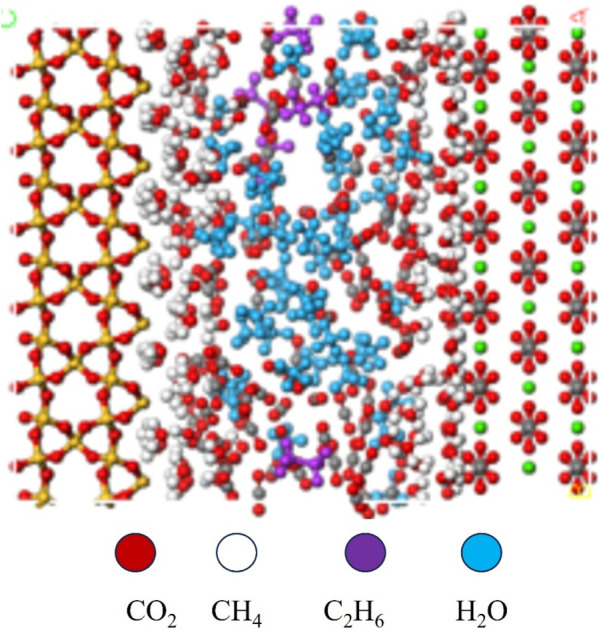

## 1. Introduction

As industrialization accelerates, the concentration of CO_2_ in the atmosphere has been continuously rising, and the global warming problem caused by this has become one of the major challenges facing human society.^1,2^ To address this crisis, the international community has established temperature control targets through the Paris Agreement, and China has also clearly proposed the strategic deployment of “carbon peaking and carbon neutrality”. Against this backdrop, CO_2_ capture, utilization and sequestration (CCUS) technology is regarded as a key technical path and a fallback technical guarantee for achieving large-scale carbon reduction.^3,4^ As an important part of CCUS technology, geological sequestration of CO_2_ involves injecting CO_2_ captured from industrial sources into deep geological reservoirs and achieving permanent sequestration through various mechanisms such as free, adsorption, dissolution and mineralization.^5^ Traditional sequestration sites mainly include deep saline aquifers, depleted oil and gas reservoirs and deep coal seams.^6,7^ However, recent studies have shown that shale gas reservoirs, with unique micro-pore structures and wide distribution, are gradually becoming a research hotspot for geological sequestration of CO_2_.^8^ Furthermore, pores are classified into micropores (*d* < 2 nm), mesopores (2 nm < *d* < 50 nm) and macropores (*d* > 50 nm), and shale gas is primarily stored in shale matrix micropores.^9,10^ Shale reservoirs have characteristics such as well-developed nanoscale pores, large specific surface area and strong adsorption capacity.^11^ It can not only fix CO_2_ through physical adsorption but also undergo geochemical reactions with CO_2_ to form calcite mineral precipitates, thereby achieving more stable mineralization sequestration.^12,13^

As a key unconventional natural gas resource, shale gas is assuming a progressively more important role within the global energy landscape.^14^ The efficient development of shale gas, when accelerated, holds great strategic significance in terms of securing energy supply and restructuring the energy mix.^15^ Conventional depletion development is difficult to effectively utilize this adsorbed gas resource, resulting in generally low recovery rates.^16^ Therefore, how to extract and utilize the adsorbed gas has become a key scientific issue for improving the recovery rate of shale gas. The CO_2_-enhanced shale gas recovery technology has emerged as the times require. Its core concept is to inject CO_2_ into the shale reservoir, using the competitive adsorption mechanism between CO_2_ and CH_4_ on the mineral surface to displace and desorb the adsorbed CH_4_ into the free state. Then, CO_2_ drives the production of free shale gas, ultimately achieving the synergistic benefits of shale gas production increase and CO_2_ geological sequestration.^17,18^ This technology not only meets China's strategic needs for “carbon peak and carbon neutrality”, but also opens up a new path for the green and efficient development of shale gas.^19^

In recent years, many researchers have conducted extensive studies on the interaction mechanism of CO_2_ sequestration and CO_2_-ESGR technology, and have made a series of significant achievements. Furthermore, recent molecular simulation studies adopting GCMC and MD methods have systematically explored gas adsorption and displacement within shale and porous carbon nanopores.^20^ Some works focused on biomass carbon substrates to reveal how N/O functional groups regulate CO_2_ physisorption, while others utilized triphenylene to characterize methane desorption by CO_2_/N_2_.^21^ Additional research tracked nanoscale water film evolution in carbonate rocks, and ternary mixture simulations further clarified moisture-driven competitive adsorption of CO_2_ and H_2_S *via* density profile and isosteric heat (*Q*_st_) analysis.^22,23^ Zhang *et al.* constructed a shale composite slit model consisting of inorganic mineral layers and organic matter layers, and used the GCMC method to investigate the influence of pore size on the effect of CO_2_ on the displacement of CH_4_.^24^ The study revealed that the CO_2_ displacement efficiency of CH_4_ was negatively correlated with the pore size of shale. The larger the pore size, the weaker the adsorption effect of the pore wall surface on CO_2_, and thus its displacement efficiency will also decrease accordingly. Xue *et al.* discovered that CO_2_ can effectively replace the octane adsorbed on the surface of calcite.^25^ Most existing studies explored CO_2_ adsorption on individual shale minerals and confirmed the strong carbon sequestration potential of carbonate rocks. However, few investigations focused on multi-mineral interfacial effects and different water contents on CO_2_ displacement of shale gas. To fill the above research gap, this study investigated the CO_2_ adsorption behavior and CO_2_ displacement of shale gas in the quartz–calcite composite structure under different water content conditions. Additionally, using DFT, the results of adsorption energy and differential charge density were calculated to elucidate the microscopic adsorption mechanisms related to charge transfer of CO_2_, CH_4_, C_2_H_6_ and H_2_O. This work is expected to provide a reliable theoretical reference for enhancing CO_2_ recovery and geological sequestration of shale gas in actual shale reservoirs containing multiple minerals.

Based on the above research background, this chapter selects quartz and calcite, which are highly abundant in the marine shale of the Longmaxi Formation in southern Sichuan, as representative minerals. A quartz–calcite composite nanopore model is constructed, and multi-scale molecular simulation methods such as GCMC, MD and DFT are used to systematically study the adsorption behavior of CO_2_ in this composite system. The focus is on the adsorption differences of CO_2_ on the surfaces of quartz and calcite under dry conditions, revealing the asymmetric distribution characteristics of CO_2_ in the composite pores. At the same time, CH_4_ and C_2_H_6_ are further introduced as representative components of shale gas, and an initial model containing shale gas is constructed to systematically study the microscopic mechanism of CO_2_ displacing shale gas in the quartz–calcite composite system. Additionally, the influence of different water contents (6 wt% to 15 wt%) on the efficiency of CO_2_ displacement is also explored, clarifying the intervention mechanism of water molecules in the competitive adsorption of gases on the surface of composite minerals. The research results aim to deepen the understanding of the CO_2_ sequestration mechanism and the CO_2_-ESGR mechanism in shale reservoirs at the molecular level, providing theoretical basis and scientific support for the development of CO_2_ displacement and enhanced sequestration technology in shale gas reservoirs.

## 2. Simulation methods

### 2.1 Model construction

**Fig. 1:**
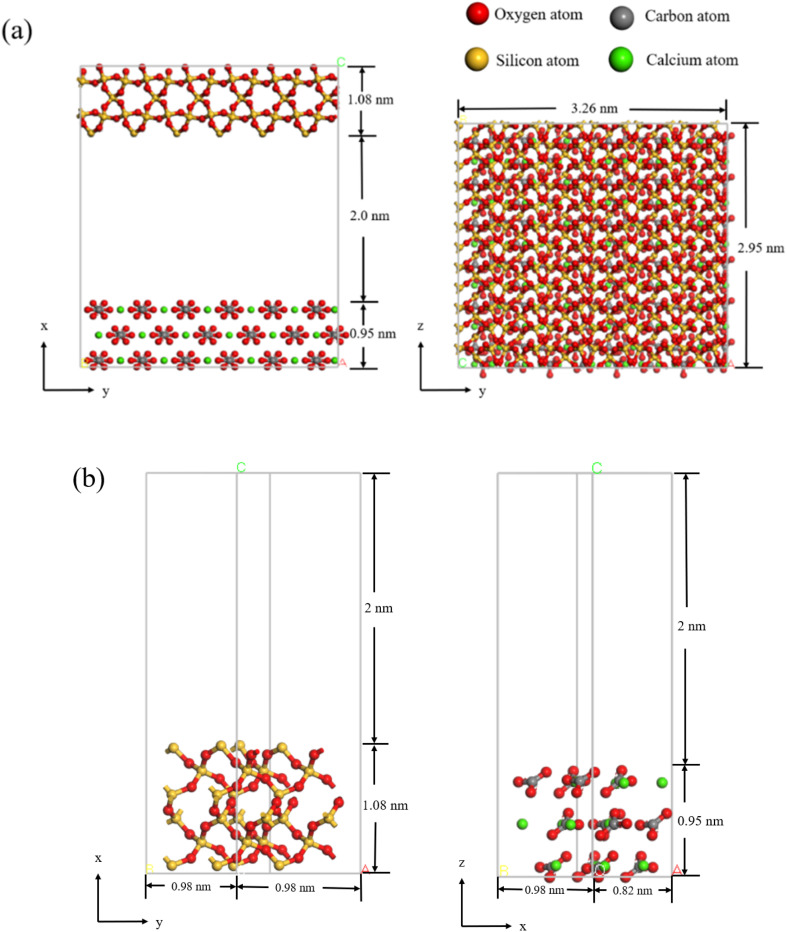
(a) Side view (left) and top view (right) of the quartz–calcite nanopore model. (b) Used for DFT calculation of quartz (001) (left) and calcite (104) (right) surface models.

The Sichuan Basin hosts the Longmashi Formation shale, which is predominantly made up of siliceous rocks and represents an important shale gas reservoir in southern China.^26^ Zhang *et al.* have selected 23 samples from 2535 m ∼2575 m of the well W202 in Weiyuan area for XRD analysis.^27^ The mineral components of the marine Longmaxi Formation shale mainly consist of quartz, calcite, and clay. The quartz content in the well W202 ranges from 40.14% to 86.37%, with an average of 61.25%; the calcite mineral content ranges from 1.52% to 16.84%, with an average of 7.12%. In this paper, quartz and calcite are selected as the main research objects. Based on previous research, the thermodynamically stable SiO_2_ (001) crystal plane^28,29^ and CaCO_3_ (104) crystal plane^30,31^ are selected to construct periodic supercell structures. After that, a 2 nm wide nanopore is constructed for the adsorption study of CO_2_. The size of the simulation system is 3.26 nm × 2.95 nm × 4.03 nm, with quartz on the top layer and calcite on the bottom layer, as shown in Fig. 1(a). At the same time, quartz and calcite simulation boxes of 0.98 nm × 0.98 nm × 3.08 nm and 0.98 nm × 0.82 nm × 2.95 nm are constructed respectively for DFT simulation calculations, as shown in Fig. 1(b).

### 2.2 Simulation details

All simulations in this research were performed using the COMPASS III force field in Materials Studio 2020. All GCMC-related simulation tasks are performed *via* the Sorption module. The Ewald method is adopted to compute long-range electrostatic interactions, whereas the atom-based method is used for van der Waals forces.^32^ The simulation is performed under constant pressure conditions, with 3 × 10^6^ steps for the equilibration phase and 4 × 10^6^ steps for the production phase.^33^ Furthermore, within NVT Monte Carlo simulations, chemical potentials are acquired using the Widom insertion method, and the Peng–Robinson equation of state (PR-EOS) calculates bulk mixture densities dependent on pressure and temperature.^34^ Then, the specific probabilities of different trial moves to exchange = 2.000, rotation = 1.000, translation = 1.000 and regrowth = 0.1000, respectively. A time step of 1.0 fs and a total simulation time of 5000 ps are applied, with the NVT ensemble adopted throughout the entire MD process. The Forcite module handles all such simulations,^35^ as shown in Table 1. The temperature and pressure control achieved through the Nose–Hoover thermostat. Additionally, self-consistent DFT calculations with unrestricted spin were performed using the DMol3 module. Electronic exchange and correlation effects are described using the PBE functional in the GGA.^36^ To achieve reliable and efficient calculations, the ECP and DNP basis set schemes are utilized.^37^ The geometric optimization applies a 3 × 3 × 1 *k*-point grid, and the convergence criteria are defined as follows: energy within 2 × 10^−5^ Ha, force within 4 × 10^−3^ Ha Å^−1^, and maximum displacement within 5 × 10^−3^ Å, as shown in Table 2.

**Table 1:** Input parameters for GCMC and MD

Input parameters	Methods, conditions and values	
	GCMC simulation	MD simulation
Temperature/K	313.15 K, 333.15 K, 353.15 K, 373.15 K	333.15 K
Pore size/nm	2 nm	
Pressure/MPa	1–100 MPa	1, 5, 10, 20, 30, and 60 MPa
Ensemble	NVT	
Forcefield	COMPASS III	
Charges	Forcefield assigned	
Summation method	Electrostatic, Ewald, vander Waals: Atombased	
Cutoff distance/Å	12.5 Å	
Time step		1.0 fs
Total simulation time		5000 ps
Equilibration steps	3 × 10^6^	
Production steps	4 × 10^6^	

**Table 2:** Input parameters for DFT

Input parameters	Methods, conditions and values
	DFT simulation
Functional	GGA PBE
Basis set	DNP
Core treatment	ECP
*k*-point set	3 × 3 × 1
Convergence criteria	2 × 10^−5^ Ha, 4 × 10^−3^ Ha Å^−1^, 5 × 10^−3^ Å

## 3. Results and discussion

### 3.1 Study on the adsorption and sequestration of CO_2_ in the quartz–calcite composite system

**Fig. 2:**
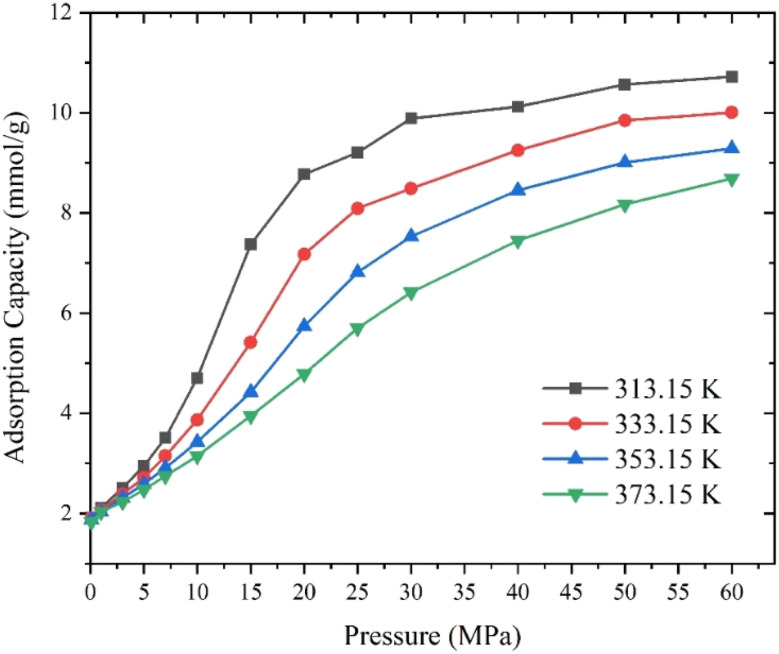
CO_2_ adsorption isotherms in quartz–calcite nanopores *versus* temperature.

In high-pressure adsorption research for geological storage, absolute adsorption refers to the total gas molecules bound to the adsorbent surface, while excess adsorption denotes the extra gas quantity relative to bulk gas at identical conditions; we adopt excess adsorption for its experimental measurability, ensuring both scientific rigor and practical applicability. The simplest model for predicting multi-component adsorption isotherms is the extended Langmuir equation.^38^ The equation representing the adsorption capacity (*V*) is as follows:1
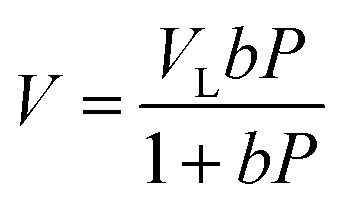
where *V*_L_ and *b* denote the maximal adsorption capacity and Langmuir constant for pure gas, respectively, *P* stands for the equilibrium pressure of the pure component. The adsorption isotherm of CO_2_ in the quartz–calcite nanopores is shown in Fig. 2. In general, as the pressure increases, the CO_2_ adsorption capacity shows an increasing trend; while as the temperature rises, the CO_2_ of adsorption capacity decreases. However, compared with the pure quartz system and the pure calcite system, the adsorption behavior of CO_2_ shows significant differences.^39,40^ In the low-pressure range (1–10 MPa), the growth of the adsorption capacity of CO_2_ is relatively slow, and it increases rapidly after reaching 10 MPa. In contrast, in the pure quartz system and the pure calcite system, the adsorption capacity of CO_2_ reaches the saturated adsorption value around 30 MPa, while in the quartz–calcite system, the adsorption capacity of CO_2_ still shows a slow increase trend even under high-pressure conditions, and does not show obvious saturation characteristics until 60 MPa. This is in agreement with the results of other experiments.^41,42^ Furthermore, micropores generate overlapping van der Waals potential between opposite pore walls, providing abundant high-affinity adsorption sites and dominating the equilibrium adsorption capacity of CO_2_.

**Fig. 3:**
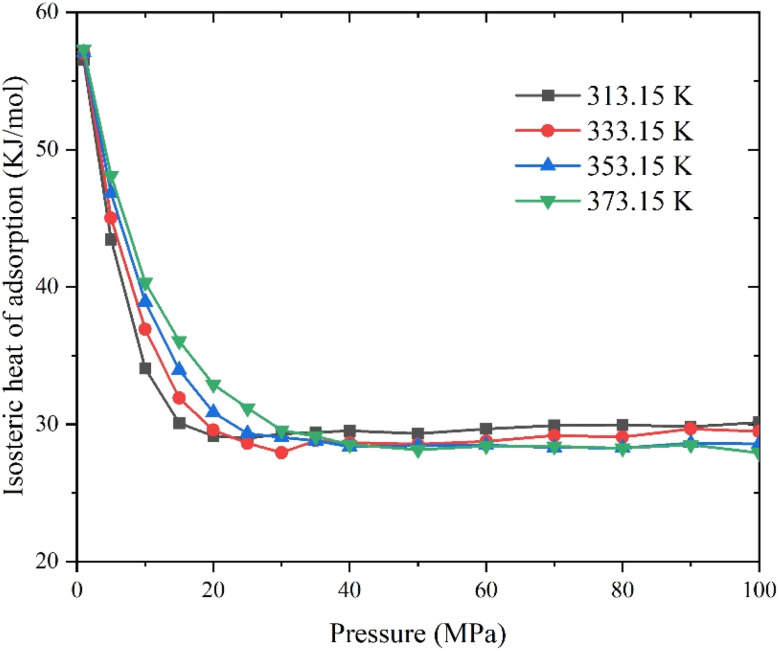
*Q*_st_ of CO_2_ in quartz–calcite composite nanopores.

Defined as the thermal effect of adsorbate binding to adsorbents, the *Q*_st_ was utilized to reveal the discrepant adsorption features of CH_4_ and CO_2_ in quartz–calcite composite nanopores. The Clausius–Clapeyron equation was applied in our molecular simulations to obtain the *Q*_st_ values. The heat effect during the adsorption process is described by the *Q*_st_, and the specific calculation approach for *Q*_st_ is as follows:^43^2
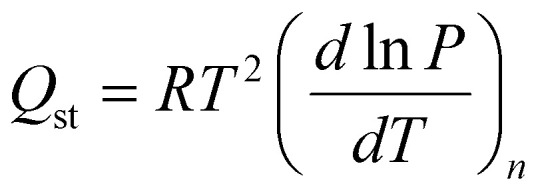
where *P* denotes the equilibrium adsorption pressure, while *T* represents the temperature respectively. Fig. 3 depicts the *Q*_st_ of CO_2_ adsorbed in quartz–calcite composite nanopores as a function of pressure under different temperatures. At pressures below 30 MPa, the *Q*_st_ value rises with increasing temperature. This can be attributed to the enhanced kinetic mobility of CO_2_ molecules at higher temperatures, which enables gas molecules to more readily access and occupy high-energy adsorption sites on mineral surfaces, thereby strengthening the interfacial adsorption interaction. At low pressures, CO_2_ molecules preferentially bind to the most energetically favorable active sites. Progressive pressure elevation gradually saturates these high-energy sites, and subsequent CO_2_ molecules can only adsorb onto low-energy sites with weaker intermolecular interactions, resulting in a rapid decrease in *Q*_st_ and a gradual stabilization.

**Fig. 4:**
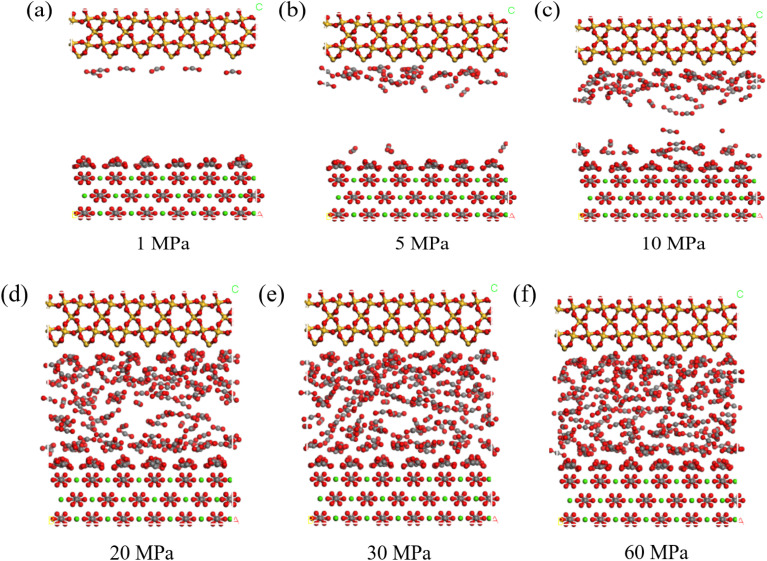
Snapshots of the CO_2_ in quartz–calcite nanopores at 333.15 K with pressure of: (a) 1, (b) 5, (c) 10, (d) 20, (e) 30, and (f) 60 MPa.

Shown in Fig. 4 is the equilibrium snapshot of CO_2_ adsorption in quartz–calcite nanopores. It is observed that CO_2_ tends to accumulate on the pore surface. At low pressures (1–10 MPa), a compact adsorption layer develops on that surface. Once the pressure hits 10 MPa, the surface adsorption layer approaches saturation. Subsequent pressure rises produce only a slight change in the density of the surface layer, whereas the density at the pore center is more significantly affected. From the snapshot, one can also infer that within the quartz–calcite composite, CO_2_ exhibits a higher affinity for calcite surfaces than for quartz surfaces.

**Fig. 5:**
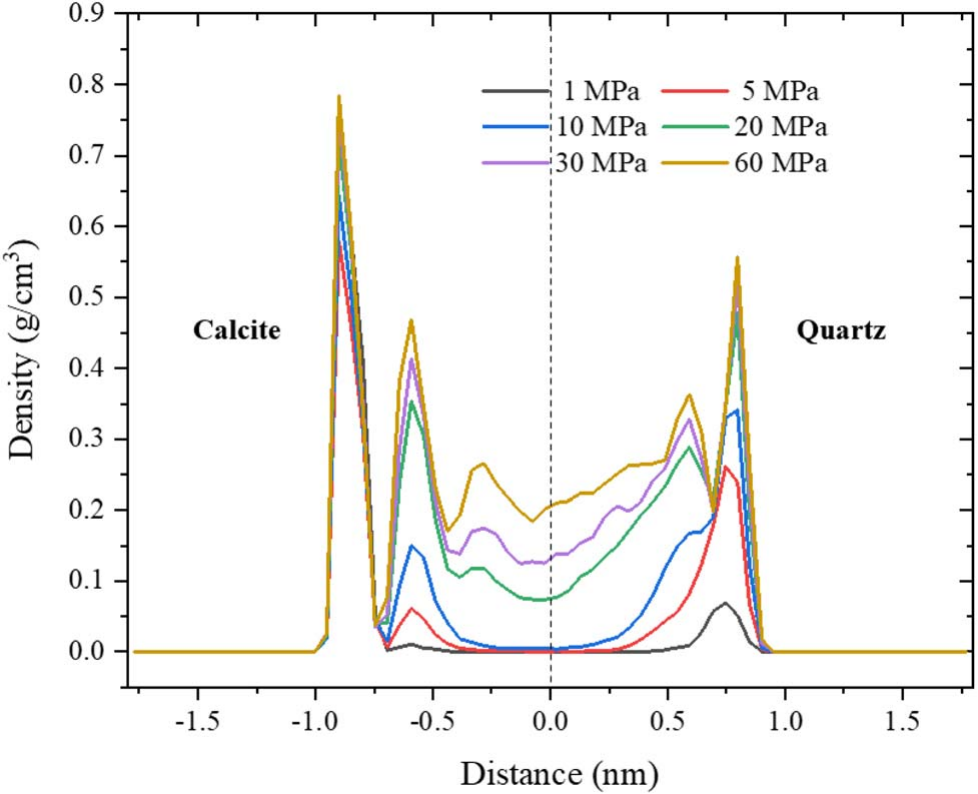
Density profile of CO_2_ in quartz–calcite nanopores at 333.15 K.

To better visualize the disparity in CO_2_ adsorption between the two sides of quartz–calcite nanopores, density profiles of CO_2_ are displayed in Fig. 5. The results indicate that, unlike the symmetric distributions observed in pure quartz or pure calcite systems, the CO_2_ distribution within the mixed-composition nanopores is asymmetric. Specifically, the adsorption peak on the calcite side is higher than that on the quartz side, implying that CO_2_ prefers adsorbing onto calcite surfaces. Furthermore, a second adsorption peak emerges on the calcite side at 5 MPa, whereas an obvious second peak on the quartz side does not appear until 20 MPa. As the pressure rises, the first adsorption peak increases slowly, while the second peak grows rapidly; the second adsorption layer on the calcite side remains larger than that on the quartz side. Upon reaching 30 MPa, the adsorption density stabilizes, which aligns with the adsorption isotherm findings. Further increasing the pressure to 60 MPa causes no significant change in the adsorption peaks on either surface, but the CO_2_ density in the pore center increases instead. Under identical temperature and pressure conditions, the adsorption density on calcite is markedly higher than that on quartz, indicating a stronger interaction between CO_2_ and the calcite surface compared to the quartz surface.

RDF is currently a very effective method for studying amorphous structures. It represents the radial distribution state of atoms around an arbitrary atom in the amorphous structure, which is spherical and symmetrical.^44^ The RDF, denoted as *g*(*r*) in the equation, gives the likelihood that a particle exists at a separation distance *r* from another tagged particle. The calculation formula is as follows:^45^3
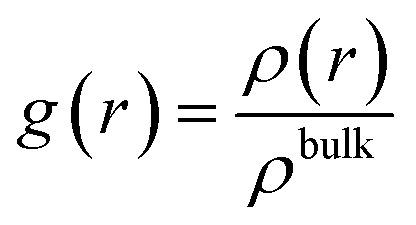


**Fig. 6:**
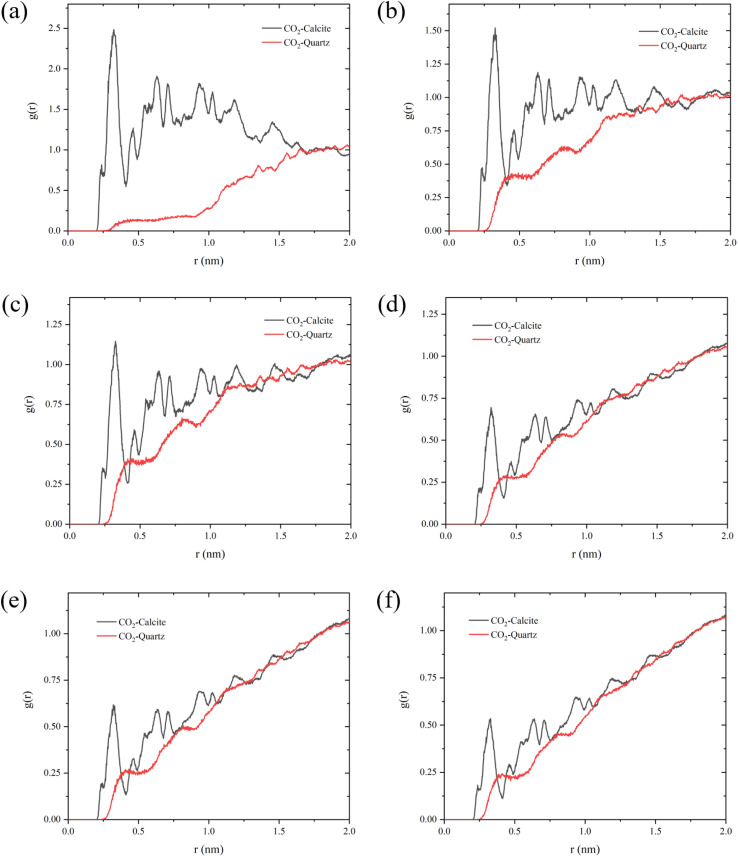
The RDF of CO_2_ in quartz–calcite nanopores at 333.15 K and varying pressures: (a) 1, (b) 5, (c) 10, (d) 20, (e) 30, and (f) 60 MPa.

The *ρ*(*r*) gives the local number density of the particles at a distance *r* from the reference substance, and *ρ*^bulk^ is the bulk density of those particles in the system. The RDF of CO_2_-calcite and CO_2_-quartz in quartz–calcite nanopores under different pressure conditions are analyzed as shown in Fig. 6. When the pressure is only 1 MPa, for the RDF curve of CO_2_-calcite, the first adsorption peak appears at 0.33 nm, which is also the maximum peak of the RDF of CO_2_-calcite, and is consistent with the density profiles results; meanwhile, the RDF curve of CO_2_-quartz does not show obvious adsorption peaks, indicating that CO_2_ mainly adsorbs near the surface of quartz. As the pressure increases, the RDF curve of CO_2_-quartz shows two adsorption peaks at 0.45 nm and 0.82 nm, indicating that there are two layers of CO_2_ adsorption layers at a distance of 0.45 nm and 0.82 nm from the surface of quartz. The RDF curve of CO_2_-calcite is always higher than that of CO_2_-quartz under different pressure, and the position of the first adsorption peak is earlier, and the number of adsorption peaks is also greater, indicating that CO_2_ is adsorbed closer to the surface of quartz and has a stronger interaction, and there are more adsorption layers.

### 3.2 Research on the displacement of shale gas by CO_2_ in the quartz–calcite composite system

**Fig. 7:**
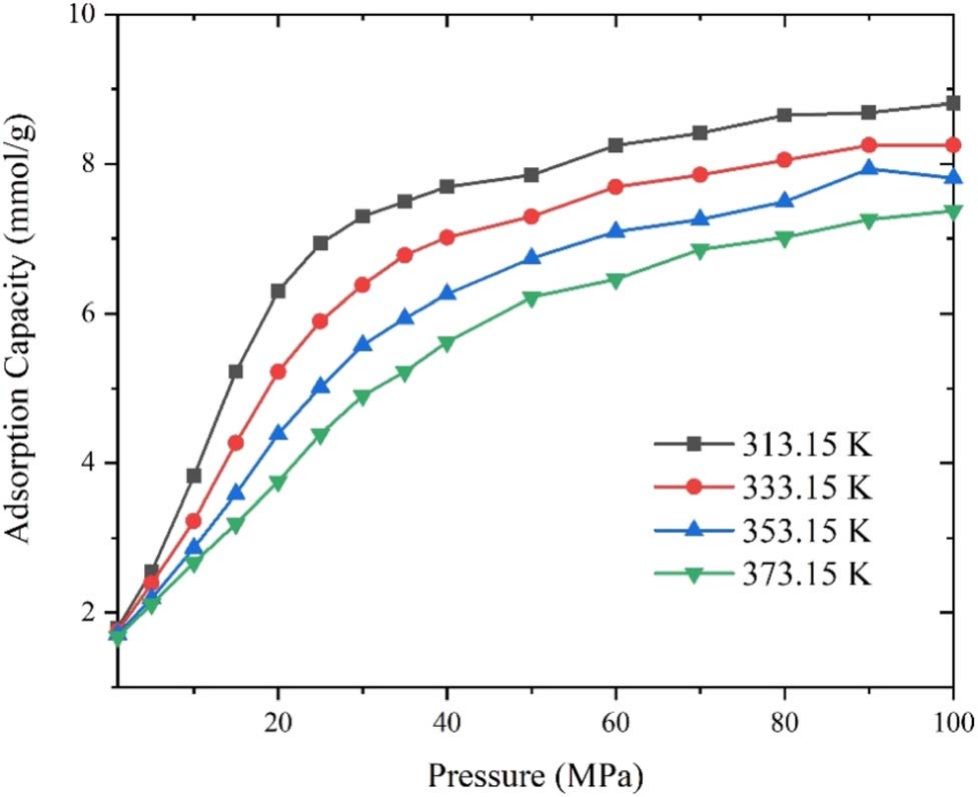
Adsorption isotherms of CO_2_ in quartz–calcite nanopores containing shale gas at different temperatures.

In order to study the ability of CO_2_ to displace shale gas, we established an initial model containing shale gas. The initial model was fixedly filled with a fixed quantity of CH_4_ and C_2_H_6_ molecules using the Sorption module, as shown in Fig. S1. This initial model was used to simulate a shale gas field containing shale gas, and the adsorption displacement of CO_2_ was carried out. The adsorption isotherms of CO_2_ were obtained through GCMC simulation, as shown in Fig. 7. The results indicate that when shale gas is present in the quartz–calcite nanopores, the adsorption isotherm of CO_2_ still conforms to the Langmuir model, and is consistent with the adsorption isotherm in the quartz–calcite nanopores without shale gas (Fig. 2). In both cases, the CO_2_'s adsorption capacity increases with the increase in pressure and decreases with the increase in temperature. However, when shale gas is present, the adsorption capacity of CO_2_ decreases. Taking 30 MPa as an example, when the temperature is 313.15 K, 333.15 K, 353.15 K and 373.15 K, the adsorption capacity of CO_2_ in the quartz–calcite nanopores without shale gas is 9.89 g cm^−3^, 8.49 g cm^−3^, 7.53 g cm^−3^ and 6.42 g cm^−3^ respectively; while with the presence of shale gas, the adsorption capacity is 7.29 g cm^−3^, 6.38 g cm^−3^, 5.58 g cm^−3^ and 4.90 g cm^−3^ respectively, reducing by 26.6%, 24.9%, 25.9% and 23.7% respectively. This indicates that the presence of shale gas reduces the adsorption capacity of CO_2_ by shale.

**Fig. 8:**
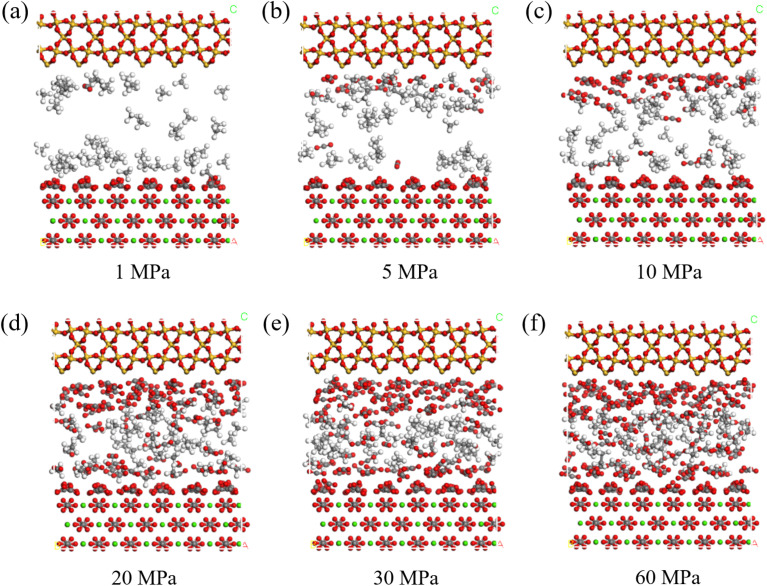
Snapshots of CO_2_ adsorption in quartz–calcite nanopores containing shale gas at 333.15 K and pressures of (a) 1, (b) 5, (c) 10, (d) 20, (e) 30 and (f) 60 MPa.

Fig. 8 presents the equilibrium snapshot of CO_2_ adsorption within quartz–calcite nanopores that contain shale gas. At a pressure of only 1 MPa, most CO_2_ molecules are found adsorbed on the calcite sidewall, and the adsorbed amount of CO_2_ is still lower than those of CH_4_ and C_2_H_6_ in the shale gas. When the pressure rises to 5 MPa, the quartz sidewall also begins to adsorb CO_2_. Once the pressure exceeds 10 MPa, the total adsorbed amount of CO_2_ in the quartz–calcite nanopores already surpasses that of the shale gas components. Moreover, under relatively low pressures, CO_2_ preferentially adsorbs on the calcite side. Only after the pressure continues to increase and the adsorption sites on the calcite surface become nearly saturated do CO_2_ molecules then start to adsorb onto the quartz side. Finally, when the adsorption sites on the quartz side reach saturation, CO_2_ diffuses into the middle of the pore.

**Fig. 9:**
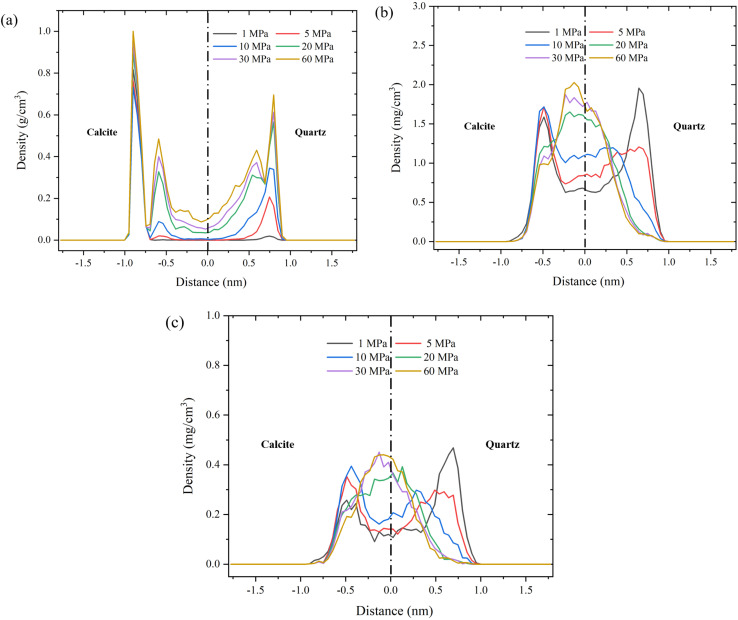
At 333.15 K, the density profiles of (a) CO_2_, (b) CH_4_ and (c) C_2_H_6_ in quartz–calcite nanopores.

Furthermore, to better distinguish the adsorption behaviors of CO_2_, CH_4_, and C_2_H_6_ within the quartz–calcite nanopores, the corresponding density profiles are computed and examined, as shown in Fig. 9. The results indicate that due to the different interactions with the quartz surface and the calcite surface, the distributions of CO_2_, CH_4_, and C_2_H_6_ in the nanopores are all asymmetric. When the pressure is 1 MPa, CO_2_ only has an adsorption peak on the left side, indicating that almost all of CO_2_ is adsorbed on the calcite side. At this time, both CH_4_ and C_2_H_6_ have higher adsorption peaks on the right side, which means that CO_2_ occupies the adsorption sites on the calcite surface, causing CH_4_ and C_2_H_6_ to be more adsorbed on the quartz surface. When the pressure reaches 5 MPa, a clear adsorption peak of CO_2_ appears on the right side, indicating that it is adsorbed on the quartz surface, resulting in a decrease in the adsorption peaks of CH_4_ and C_2_H_6_. As the pressure goes to 20 MPa, a second adsorption peak of CO_2_ appears at a distance of 0.41 nm from the calcite surface, which is exactly the position of the peak on the left side of CH_4_ and C_2_H_6_ in the low-pressure condition. Therefore, after 20 MPa, the adsorption peaks on both sides of CH_4_ and C_2_H_6_ disappear, and the density profiles is concentrated in the middle position of the pore. As the pressure rises to 30 MPa and 60 MPa, the differences in the adsorption density of CO_2_ gradually become more obvious. The adsorption peak on the left side is higher than that on the right side. In summary, compared with CH_4_ and C_2_H_6_, CO_2_ shows a preference for adsorption onto calcite surfaces at lower pressures. In contrast, CH_4_ and C_2_H_6_ are confined to the quartz surface or the secondary adsorption layer away from calcite, because the calcite adsorption sites are already occupied by CO_2_. As pressure increases, the CO_2_ uptake gradually rises, and its density profiles become more concentrated near the shale surface. Meanwhile, the density profiles of CH_4_ and C_2_H_6_ are localized in the pore center. This is mainly because CO_2_ with a smaller dynamic diameter can enter the narrow pores, while CH_4_ and C_2_H_6_, due to their larger molecular volumes and significant steric hindrance effects, have significantly lower saturated adsorption capacities compared to CO_2_. At the same time, CO_2_ molecules have a stronger linear quadrupole moment, which enables them to have stronger interactions with the mineral surface.^46^ This results in significant differences in adsorption among CO_2_, CH_4_ and C_2_H_6_.

**Fig. 10:**
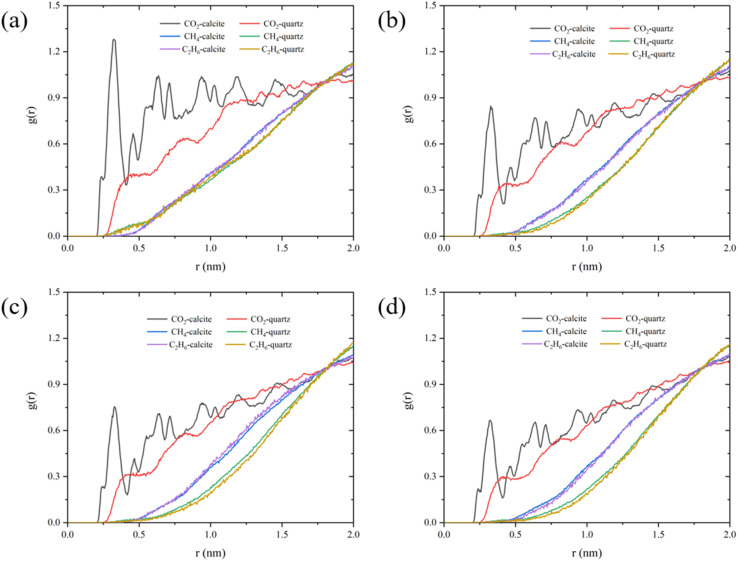
RDF of CO_2_, CH_4_, and C_2_H_6_ in quartz–calcite nanopores at 333.15 K with varying pressure: (a) 10, (b) 20, (c) 30, and (d) 60 MPa.

Fig. 10 shows the RDF of CO_2_, CH_4_ and C_2_H_6_ respectively on the surface of calcite and quartz. It can be seen that under the four different pressures, the RDF curves of CH_4_ and C_2_H_6_ on the two sides of the quartz–calcite nanopores are consistent in trend, both tending to move away from the shale surface. This means that when CO_2_ is present, the farther away from the quartz surface and calcite surface, the greater the density of CH_4_ and C_2_H_6_. Thus, after CO_2_ saturates the shale surface adsorption sites, CH_4_ and C_2_H_6_ are forced into the pore center and show no clear peaks. The CO_2_-calcite RDF first peak is positioned earlier, is more distinct, and has a greater magnitude than that of CO_2_-quartz, confirming that CO_2_ favors calcite over quartz surfaces.

Additionally, the diffusion behavior of CO_2_, CH_4_, and C_2_H_6_ was investigated. In quartz–calcite nanopores, gas diffusion properties are commonly characterized using the MSD, which is defined by the following formula:^47^4
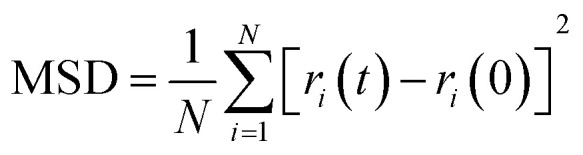


The adsorbent amount is given by *N*, and the positions at time *t* and initially are denoted as *r*_*i*_(*t*) and *r*_*i*_(0), respectively. It should be noted that MSD and the diffusion coefficient (*D*) are closely associated. The value of *D* is 1/6 of the slope of MSD:^48^5
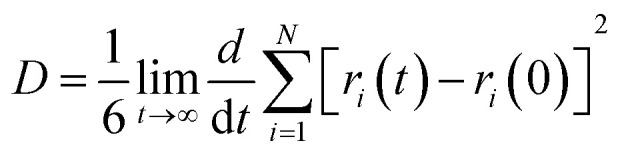


**Fig. 11:**
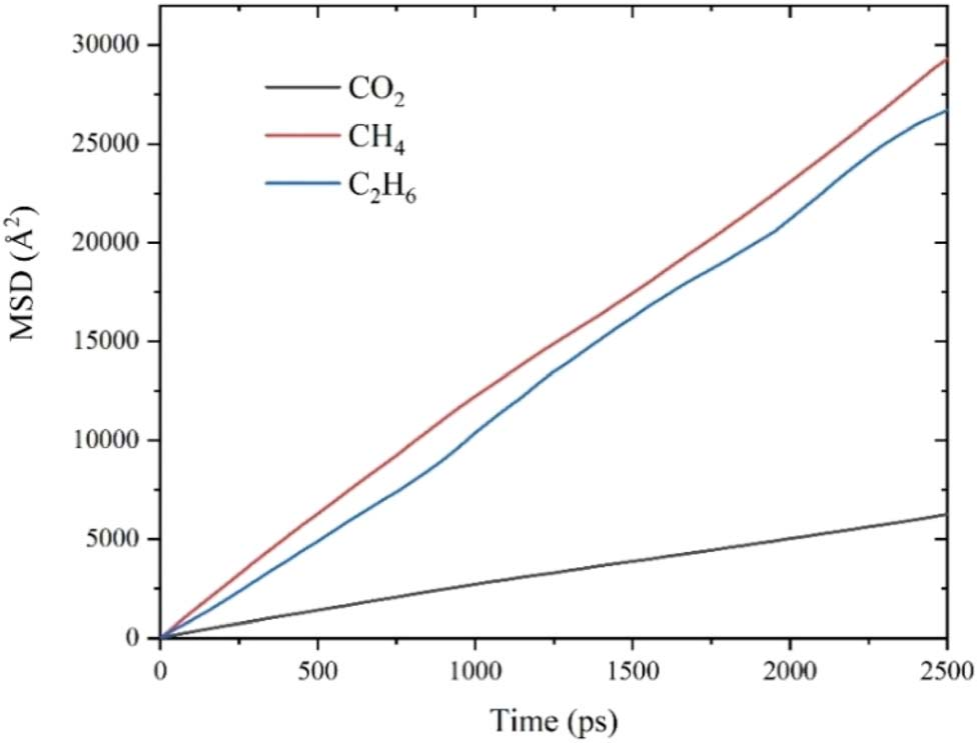
The MSD of gases in quartz–calcite nanopores at 333.15 K and 30 MPa.

Fig. 11 shows the MSD of CO_2_, CH_4_ and C_2_H_6_ in quartz–calcite nanopores containing shale gas under dry conditions. At this time, the diffusion coefficients *D* of CO_2_, CH_4_ and C_2_H_6_ are 0.42 Å^2^ ps^−1^, 1.95 Å^2^ ps^−1^ and 1.78 Å^2^ ps^−1^. It can be seen that the diffusion coefficient of CO_2_ molecules is smaller than that of CH_4_ and C_2_H_6_ molecules, which means that in the quartz–calcite nanopores containing shale gas, the interaction between CO_2_ and the two sides of the shale is the strongest and the adsorption is the most stable; while the larger diffusion coefficients of CH_4_ and C_2_H_6_ indicate that the adsorption of these two shale gases on the two sides of the quartz–calcite nanopores is weak, and they have stronger diffusion properties in the shale and are more easily displaced by CO_2_.

### 3.3 Effect of water content on CO_2_ sequestration and displacement of shale gas

Water content is a key factor affecting CO_2_ storage and shale gas displacement in real shale reservoirs. To examine its impact on CO_2_ displacement, composite quartz–calcite nanopore models with 6, 10, 12, and 15 wt% water content are built (Fig. S2). In the quartz–calcite nanopore with 6 wt% water content, water molecules are sparsely adsorbed on the quartz side but densely adsorbed on the calcite side. This may be due to the hydrophobicity of quartz and the hydrophilicity of calcite. Once the level reaches 10 wt%, the majority of quartz surface adsorption sites are occupied by water, and a secondary water layer appears on the calcite surface. For water contents of 12 wt% and 15 wt% in the quartz–calcite nanopores, water molecules completely dominate the adsorption sites on both shale surfaces, thereby significantly suppressing CH_4_ and C_2_H_6_ uptake.

**Fig. 12:**
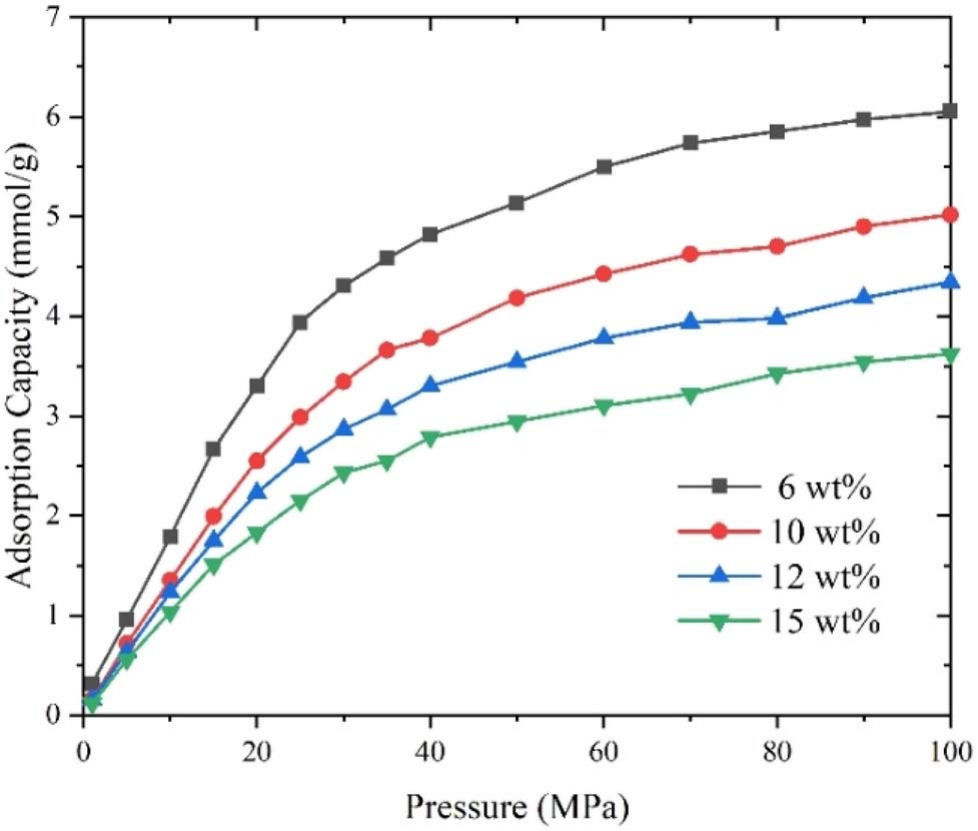
Adsorption isotherms of CO_2_ in quartz–calcite nanopores at 333.15 K with varying water content.

Fig. 12 presents how CO_2_ adsorption isotherms behave in quartz–calcite nanopores containing shale gas at different water levels. Relative to the scenario with dry shale gas, increasing the water content leads to a marked reduction in the CO_2_ adsorption capacity. Taking a pressure of 30 MPa as an example, the adsorption capacitys of CO_2_ in quartz–calcite nanopores containing shale gas with moisture contents of 6 wt%, 10 wt%, 12 wt%, and 15 wt% are 4.30 mmol g^−1^, 3.35 mmol g^−1^, 2.87 mmol g^−1^, and 2.43 mmol g^−1^, respectively. Compared with the 6.38 mmol g^−1^ of CO_2_ adsorption in the dry quartz–calcite nanopores containing shale gas, the adsorption capacitys of CO_2_ in the water-containing shale gas quartz–calcite nanopores decrease by 32.5%, 47.5%, 55.0%, and 61.9%, respectively. The results indicate that when water is present, the adsorption capacity of CO_2_ significantly decreases, and the greater the water content, the greater the degree of decrease. Under the same water-containing conditions, the adsorption capacitys of CO_2_ in quartz–calcite nanopores without shale gas are 6.69 mmol g^−1^ (6 wt%), 5.62 mmol g^−1^ (10 wt%), 5.06 mmol g^−1^ (12 wt%), and 4.38 mmol g^−1^ (15 wt%), respectively. The presence of shale gas leads to a reduction of 35.7%, 40.4%, 43.3%, and 44.5% in the adsorption capacity of CO_2_. The above data indicate that a higher moisture content has a negative impact on the CO_2_ displacement and replacement of shale gas, and the presence of shale gas also affects the adsorption capacity of CO_2_, thereby affecting the efficiency of CO_2_ displacement of shale gas.

**Fig. 13:**
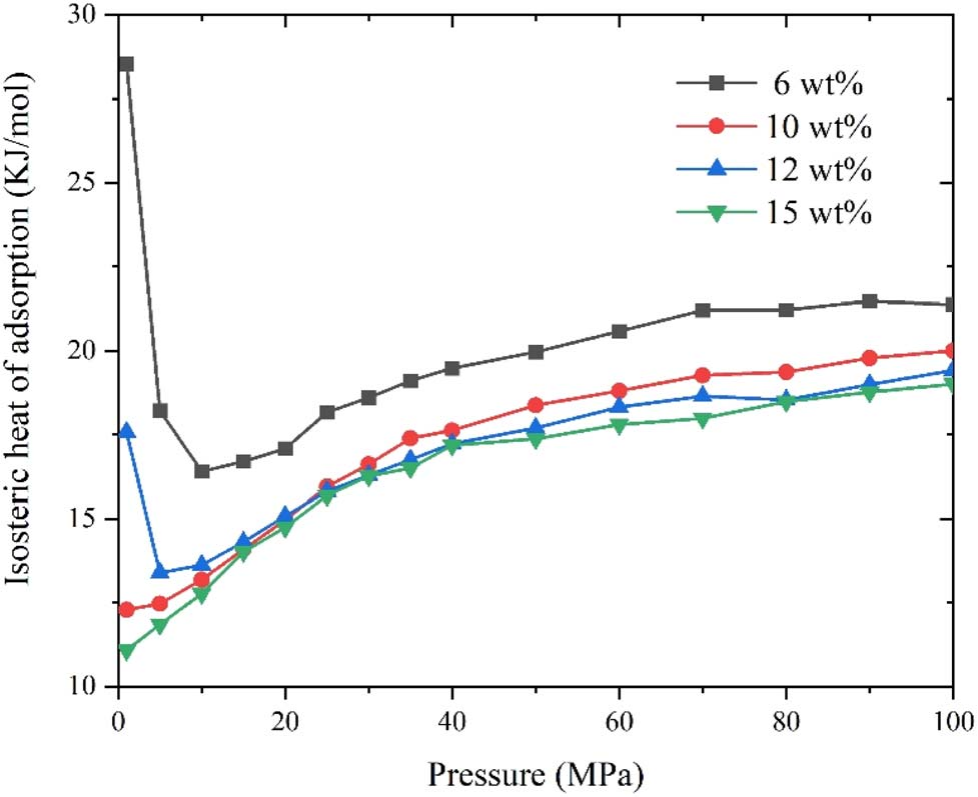
*Q*_st_ of CO_2_ in hydrated quartz–calcite composite nanopores.

Fig. 13 illustrates the variation of the *Q*_st_ of CO_2_ with pressure under four different water contents. At a same water content, *Q*_st_ declines rapidly when the pressure is below 15 MPa. Above approximately 15 MPa, however, the *Q*_st_ values gradually rebound with increasing pressure for all water contents. Moreover, the *Q*_st_ decreases progressively as the water content rises, this due to a higher amount of pore water means that more water molecules occupy the internal pore surfaces, competing for and occupying a substantial fraction of the effective adsorption sites. This occupancy weakens the van der Waals interactions between CO_2_ and the mineral surfaces, reduces the interfacial affinity for gas adsorption, and consequently leads to a lower *Q*_st_.

**Fig. 14:**
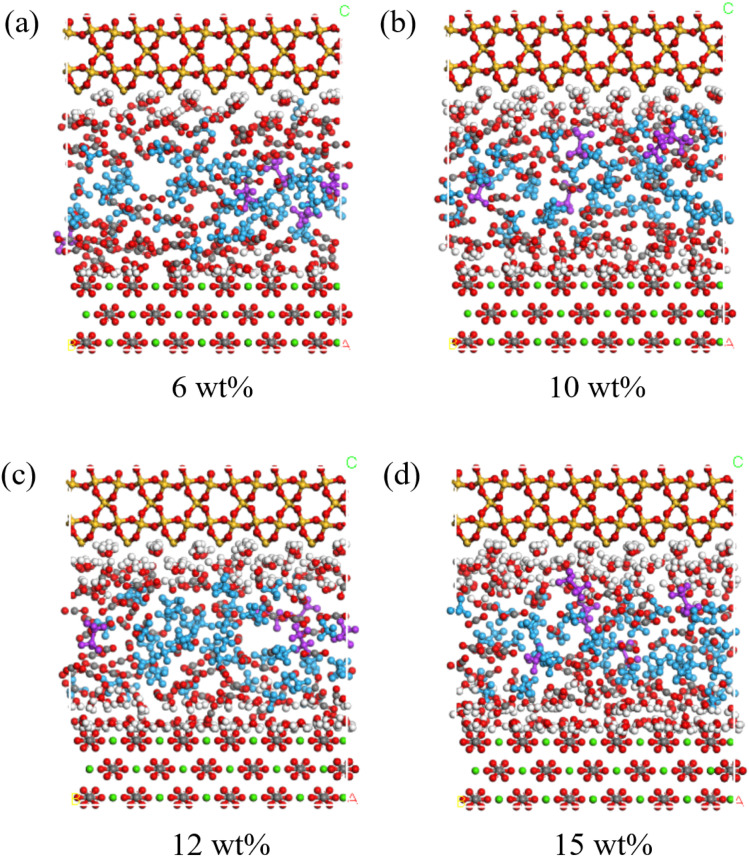
Snapshots of the CO_2_ adsorption in quartz–calcite nanopores of shale gas under conditions of 333.15 K. The water contents are (a) 6 wt%, (b) 10 wt%, (c) 12 wt%, and (d) 15 wt% (blue represents CH_4_ molecules, and purple represents C_2_H_6_ molecules).

Following molecular dynamics simulations, Fig. 14 presents snapshots of the adsorption equilibrium states of CO_2_, CH_4_, and C_2_H_6_ in the quartz–calcite composite system containing shale gas at various water contents. At a water content of 6 wt%, the low moisture level means H_2_O molecules do not fully cover the nanopore walls; some CO_2_ molecules still adsorb onto the pore surfaces, whereas CH_4_, and C_2_H_6_ remain dispersed within the pore space. When the water content rises to 10 wt%, most pore surfaces become occupied by water molecules, leading to a marked reduction in CO_2_ uptake. Upon further increasing the water content to 15 wt%, the adsorption sites on both nanopore walls are progressively and completely taken over by water molecules. Consequently, the CO_2_'s adsorbed amount drops sharply, and like CH_4_, and C_2_H_6_, CO_2_ can only be distributed in the pore center, unable to adsorb onto either the quartz or calcite surfaces.

**Fig. 15:**
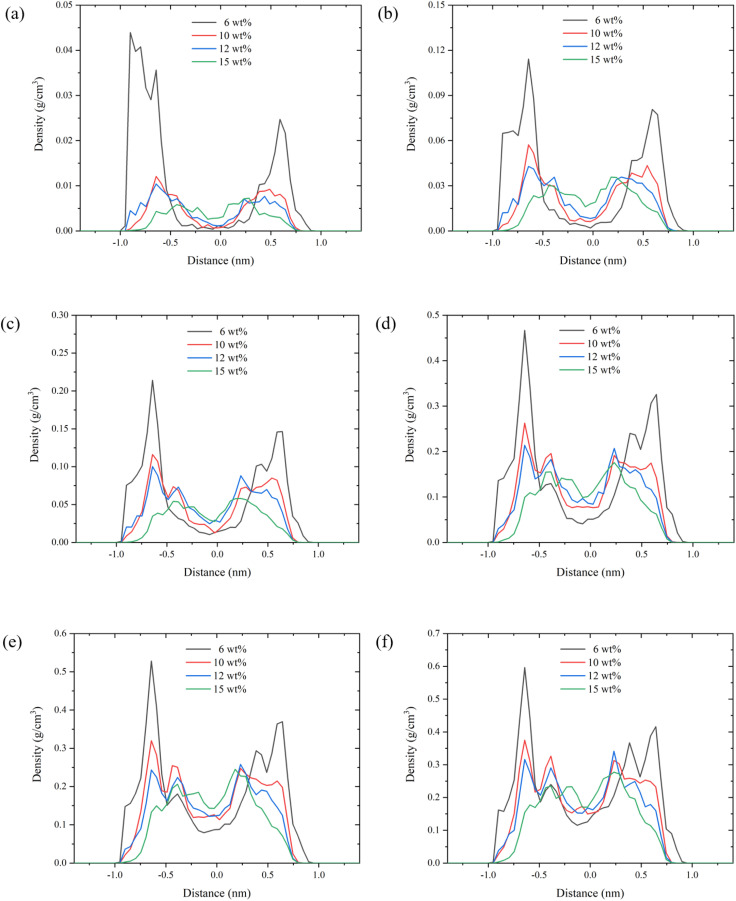
CO_2_ density profiles in quartz–calcite nanopores with water and shale gas at 333.15 K and pressures of (a) 1, (b) 5, (c) 10, (d) 20, (e) 30, and (f) 60 MPa.

Fig. 15, S3 and S4 respectively show the density profiles of CO_2_, CH_4_, and C_2_H_6_ in quartz–calcite nanopores under different water content conditions. When the water content is only 6 wt%, CO_2_ has obvious adsorption peaks on both surfaces of the nanopores, and as the pressure increases, the peaks keep increasing, even reaching a second adsorption peak at a pressure of 10 MPa; while CH_4_, and C_2_H_6_ still have relatively obvious adsorption peaks on both surfaces at 1 MPa, but when the pressure turns up to 10 MPa, the density profiles curves of both decrease completely to the middle of the pores. As the water content goes to 10 wt%, the adsorption peak of CO_2_ significantly decreases, and compared with the 6 wt% water content condition, the decrease is significant under different pressures; at this time, CH_4_, and C_2_H_6_ have no obvious formed adsorption peaks, and thereafter, as the pressure and water content change, the shape trend of their density profiles curves is consistent. Raising the water content to 15 wt% eliminates the second adsorption peak of CO_2_ under high pressure, confirming that abundant moisture impairs CO_2_ adsorption capacity. Nonetheless, in hydrated quartz–calcite nanopores, the density profiles of CO_2_ are still positioned nearer to both quartz and calcite surfaces compared with those of CH_4_ and C_2_H_6_, indicating higher CO_2_ adsorption in shale pores. In conclusion, although H_2_O molecules significantly hinder CO_2_ adsorption in the quartz–calcite composite nanopores, CO_2_ preserves its superiority in displacing CH_4_ and C_2_H_6_.

**Fig. 16:**
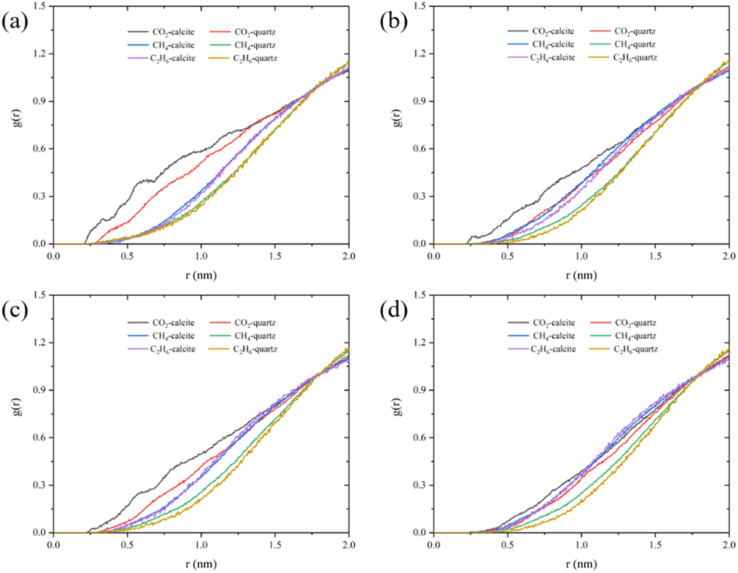
The RDF of CO_2_, CH_4_ and C_2_H_6_ in quartz–calcite nanopores under varying water contents of (a) 6 wt%, (b) 10 wt%, (c) 12 wt% and (d) 15 wt%.

Fig. 16 shows the RDF of CO_2_, CH_4_, and C_2_H_6_ with respect to the surface of calcite and quartz under different water contents. As shown in Fig. 14, under the four different water content conditions, the RDF curves of CH_4_, and C_2_H_6_ on the two sides of the quartz–calcite nanopores are consistent in trend, both tending to be away from the shale surface. This means that when there is H_2_O present, the farther away from the quartz surface and calcite surface, the greater the density of CH_4_, and C_2_H_6_, indicating that CH_4_, and C_2_H_6_ can only be distributed in the middle position of the pores away from the shale surface, and there are no formed peaks. For CO_2_, when the water content is only 6 wt%, there are still obvious differences in the peak shapes of the RDF of CO_2_ with respect to the calcite surface and quartz surface. It can be seen that the RDF of CO_2_ with respect to the calcite surface is higher than that with respect to the quartz surface, and the RDF curve of CO_2_-calcite has more obvious peaks at 0.37 nm and 0.69 nm, indicating that there are two CO_2_ adsorption layers on the calcite surface. When the water content reaches 10 wt% and 12 wt%, the shapes of the RDF curves of CO_2_ with respect to both sides are already very similar to those of CH_4_, and C_2_H_6_. At this time, the RDF curve of CO_2_-calcite is still on the upper side, indicating that when water is present, the adsorption of CO_2_ with respect to calcite is the strongest. When the water content reaches 15 wt%, the RDF shapes of CO_2_, CH_4_, and C_2_H_6_ in the quartz–calcite nanopores are consistent, and are the same as the RDF shapes of CO_2_ with respect to the calcite surface and quartz surface in Fig. 9. This indicates that water has absolute competitiveness for the adsorption sites on the calcite surface and quartz surface, and has the strongest interaction with the two sides of the quartz–calcite nanopore walls, resulting in CO_2_, CH_4_, and C_2_H_6_ being able to only be adsorbed at the center position away from the nanopore surface.

**Fig. 17:**
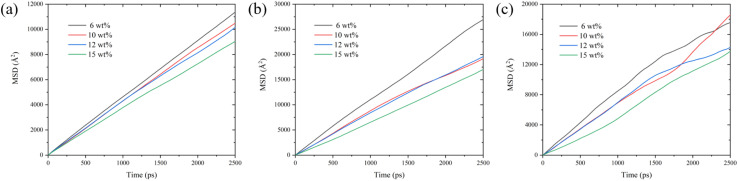
Under different water content conditions, the MSD of (a) CO_2_, (b) CH_4_, and (c) C_2_H_6_ in quartz–calcite nanopores.

Fig. 17 shows the MSD of CO_2_, CH_4_ and C_2_H_6_ in quartz–calcite nanopores with different water contents. The diffusion coefficients *D* of CO_2_ molecules at water contents of 6 wt%, 10 wt%, 12 wt% and 15 wt% are 0.76 Å^2^ ps^−1^, 0.70 Å^2^ ps^−1^, 0.68 Å^2^ ps^−1^ and 0.60 Å^2^ ps^−1^ respectively; the diffusion coefficients *D* of CH_4_ molecules at varying water contents are 1.80 Å^2^ ps^−1^, 1.28 Å^2^ ps^−1^, 1.31 Å^2^ ps^−1^ and 1.14 Å^2^ ps^−1^ respectively; the diffusion coefficients *D* of C_2_H_6_ molecules at varying water contents are 1.17 Å^2^ ps^−1^, 1.24 Å^2^ ps^−1^, 0.95 Å^2^ ps^−1^ and 0.92 Å^2^ ps^−1^ respectively. These data indicate that the CO_2_'s diffusion coefficient is smaller than that of CH_4_ and C_2_H_6_ molecules under the same water content conditions, which means that in the water-containing quartz–calcite nanopores with shale gas, apart from water molecules, CO_2_ is the most stable adsorbed on the surfaces of both sides of the shale and is least likely to be displaced; while the diffusion coefficients of CH_4_ and C_2_H_6_ are large, indicating that the adsorption of these two shale gases on the two sides of the quartz–calcite nanopores is weak, and they have stronger diffusion in the shale and are more likely to be displaced by CO_2_. Moreover, as the water level increases, the diffusion coefficients of CO_2_, CH_4_ and C_2_H_6_ all show a decreasing trend, because more and more water molecules occupy the nanopores, resulting in a reduction of the diffusion space of CO_2_, CH_4_ and C_2_H_6_, and the diffusion trend is blocked, and the diffusion coefficient decreases.

### 3.4 Adsorption of CO_2_, CH_4_, C_2_H_6_, and H_2_O by the DFT viewpoint

In order to deeply explore the microscopic mechanism of the influence of H_2_O on the adsorption of CO_2_, CH_4_ and C_2_H_6_ in quartz–calcite nanopores, the *E*_ads_ values of H_2_O, CO_2_, CH_4_ and C_2_H_6_ on the surfaces of quartz and calcite are respectively calculated. The adsorption energies (*E*_ads_) are defined as follows:^49^

6 *E*_ads_ = *E*_total_ − *E*_adsorbent_ − *E*_adsorbate_

where *E*_total_ is the total energy of adsorbents and adsorbates. *E*_adsorbent_ and *E*_adsorbate_ refer to the energy of adsorbents and adsorbates. The *E*_ads_ values of CO_2_ on quartz and calcite surfaces are −4.71 and −20.85 kJ mol^−1^ respectively, while those of CH_4_ are −3.73 and −6.02 kJ mol^−1^ independently, and those of C_2_H_6_ are −4.05 and −8.54 kJ mol^−1^, respectively. These results demonstrate the adsorption affinity follows the order: CO_2_ > C_2_H_6_ > CH_4_. All of them are greater than −40 kJ mol^−1^, declaring that CO_2_, CH_4_ and C_2_H_6_ exhibit physical adsorption characteristics on quartz and calcite surfaces.^50^ However, the *E*_ads_ values of H_2_O on quartz and calcite surfaces are −62.48 kJ mol^−1^ and −76.32 kJ mol^−1^, both of which are less than −40 kJ mol^−1^, meaning chemical adsorption. Moreover, the *E*_ads_ values of CO_2_ on quartz and calcite surfaces are more negative than those of CH_4_ and C_2_H_6_, suggesting the interaction between surfaces of quartz or calcite and CO_2_ is stronger. This finding is consistent with the GCMC simulation results, indicating the feasibility of CO_2_ displacing shale gas.

**Fig. 18:**
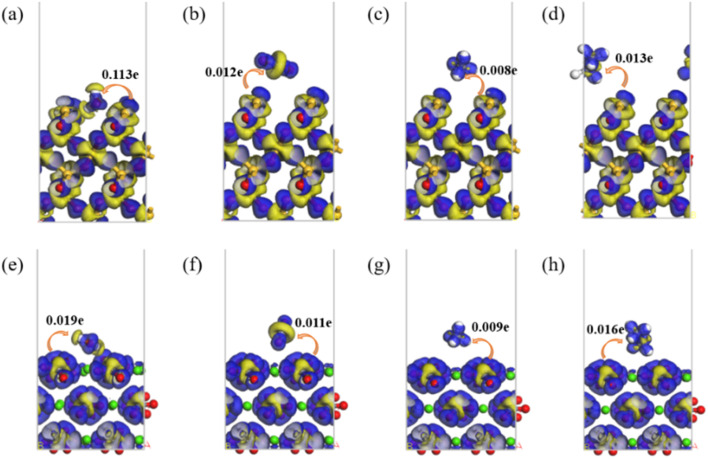
Mulliken charge and deformed charge density of (a) H_2_O-quartz, (b) CO_2_-quartz, (c) CH_4_-quartz, (d) C_2_H_6_-quartz, (e) H_2_O-calcite, (f) CO_2_-calcite, (g) CH_4_-calcite, and (h) C_2_H_6_-calcite.

To better elucidate the adsorption mechanism, Fig. 18 presents a detailed analysis of the Mulliken charges and differential charge densities for H_2_O, CO_2_, CH_4_, and C_2_H_6_ adsorbed on quartz and calcite surfaces. The Mulliken charge serves as a quantitative measure of the net charge associated with molecular adsorption. As calculated, the net charges on the quartz surface are 0.113e for H_2_O, 0.012e for CO_2_, 0.008e for CH_4_, and 0.013e for C_2_H_6_. These values indicate that, compared with CH_4_ and C_2_H_6_, CO_2_ exhibits a more pronounced electron transfer with the mineral surface, implying a greater interaction between CO_2_ and the quartz surface.^51^ On the calcite surface, the net charges of H_2_O, CO_2_, CH_4_, and C_2_H_6_ are 0.019e, 0.011e, 0.009e, and 0.016e, indicating that CO_2_ still transfers more electrons than CH_4_ on calcite. The differential charge density offers an intuitive depiction of charge distribution for these four species on quartz and calcite, with yellow and blue representing electron depletion and accumulation. A distinct electron-rich region appears between the oxygen of CO_2_ and the oxygen on the quartz surface. In contrast, no such obvious electron region is observed for CH_4_ or C_2_H_6_ on either surface, suggesting their weaker interactions with quartz and calcite. These findings reveal the microscopic mechanism underlying CO_2_ ability to displace shale gas within quartz–calcite nanopores, thereby confirming the feasibility of CO_2_-driven shale gas displacement.

## 4. Conclusions

Based on the mineral composition of the marine shale of the Longmoxi Formation in southern Sichuan, a composite nanopore model composed of quartz and calcite is constructed. Molecular simulation methods are employed to systematically study the adsorption and diffusion behaviors of CO_2_ in the nanopores of this composite system. The effects of drying conditions and different water contents (6 wt% to 15 wt%) on CO_2_ sequestration and the displacement of CH_4_ and C_2_H_6_ are also investigated in detail. In summary, in the quartz–calcite composite system of the simulated shale reservoir, CO_2_ can effectively compete for adsorption sites due to its stronger interaction with the mineral surface, and can displace CH_4_ and C_2_H_6_ to the pore center, demonstrating good potential for CO_2_-driven gas displacement. However, the presence of formation water significantly weakens the adsorption capacity and displacement efficiency of CO_2_ through the competitive adsorption mechanism. The research results show a theoretical basis for in-depth understanding of CO_2_ sequestration and CO_2_-ESGR research in shale reservoirs, and have important reference value for evaluating the CO_2_ displacement efficiency under actual reservoir conditions.

## Conflicts of interest

There are no conflicts to declare.

## Supplementary Material

RA-016-D6RA04585E-s001

## Data Availability

The authors declare that the data will be made available on request. Supplementary information (SI) is available. See DOI: https://doi.org/10.1039/d6ra04585e.
