# Supplementary material for: Molecular simulation study on CO2 sequestration and shale gas displacement in the quartz–calcite composite system
Source: RSC Adv. 2026 Jul 13;16(37):39933–49. doi: 10.1039/d6ra04585e (PMC13358895; doi:10.1039/d6ra04585e)
Supplement: Supplementary file 1 [file RA-016-D6RA04585E-s001.pdf]

Supplementary Information (SI) for RSC Advances.
This journal is © The Royal Society of Chemistry 2026

Supplementary Information

Molecular simulation study on CO_2 sequestration and shale gas displacement in the quartz-calcite composite system

Yifeng Ma, Caili Dai*, Jianwei Gu*

Shandong Key Laboratory of Oil and Gas Field Chemistry, Department of Petroleum Engineering, China University of Petroleum (East China), Qingdao 266580, China

* Corresponding author.
E-mail addresses: Caili Dai ✉ daicl@upc.edu.cn; Jianwei Gu ✉ gjwLcp@upc.edu.cn

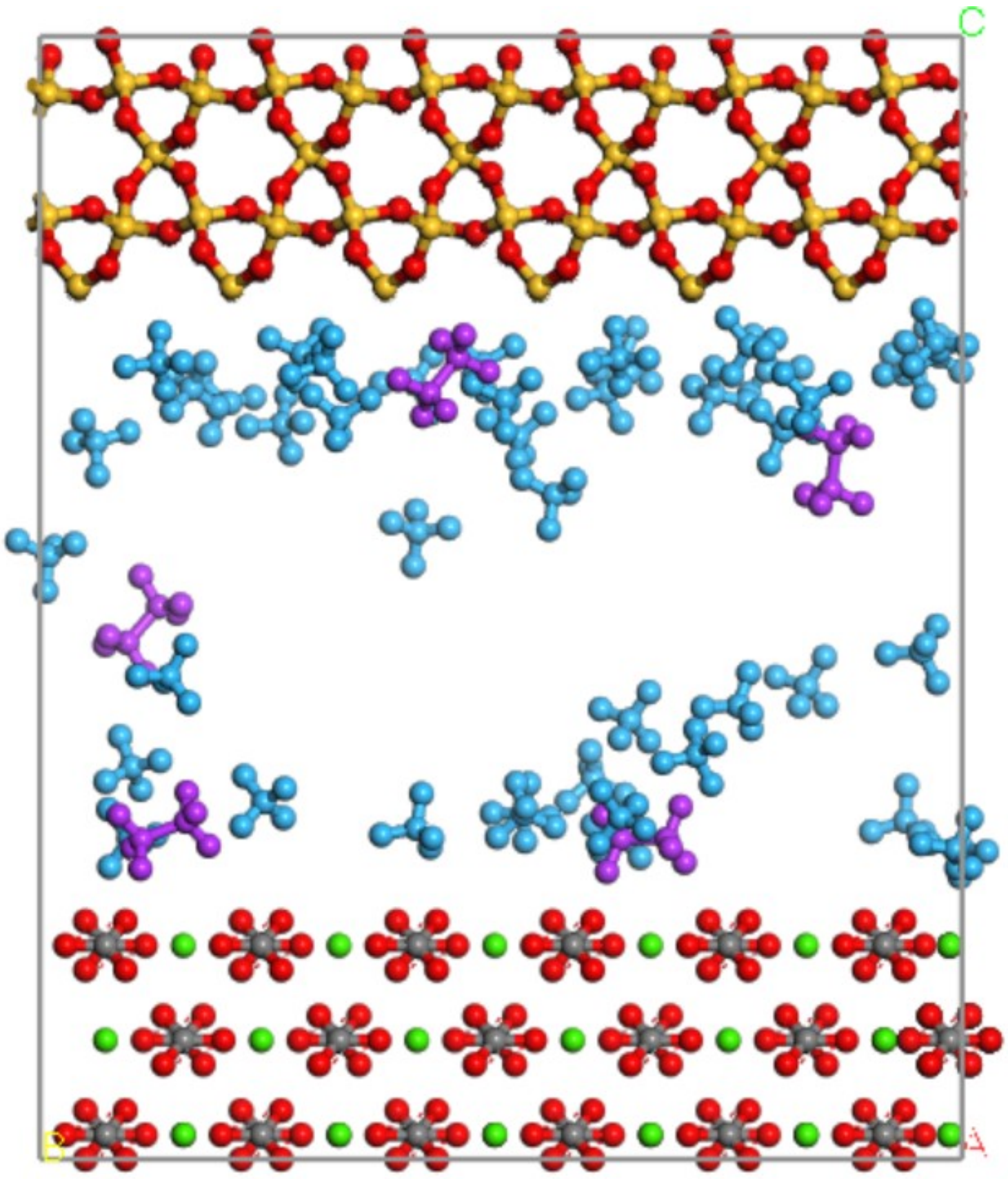

Fig. S1. A quartz-calcite composite system model containing CH_4 and C_2H_6 (blue represents CH_4 molecules, and purple represents C_2H_6 molecules).

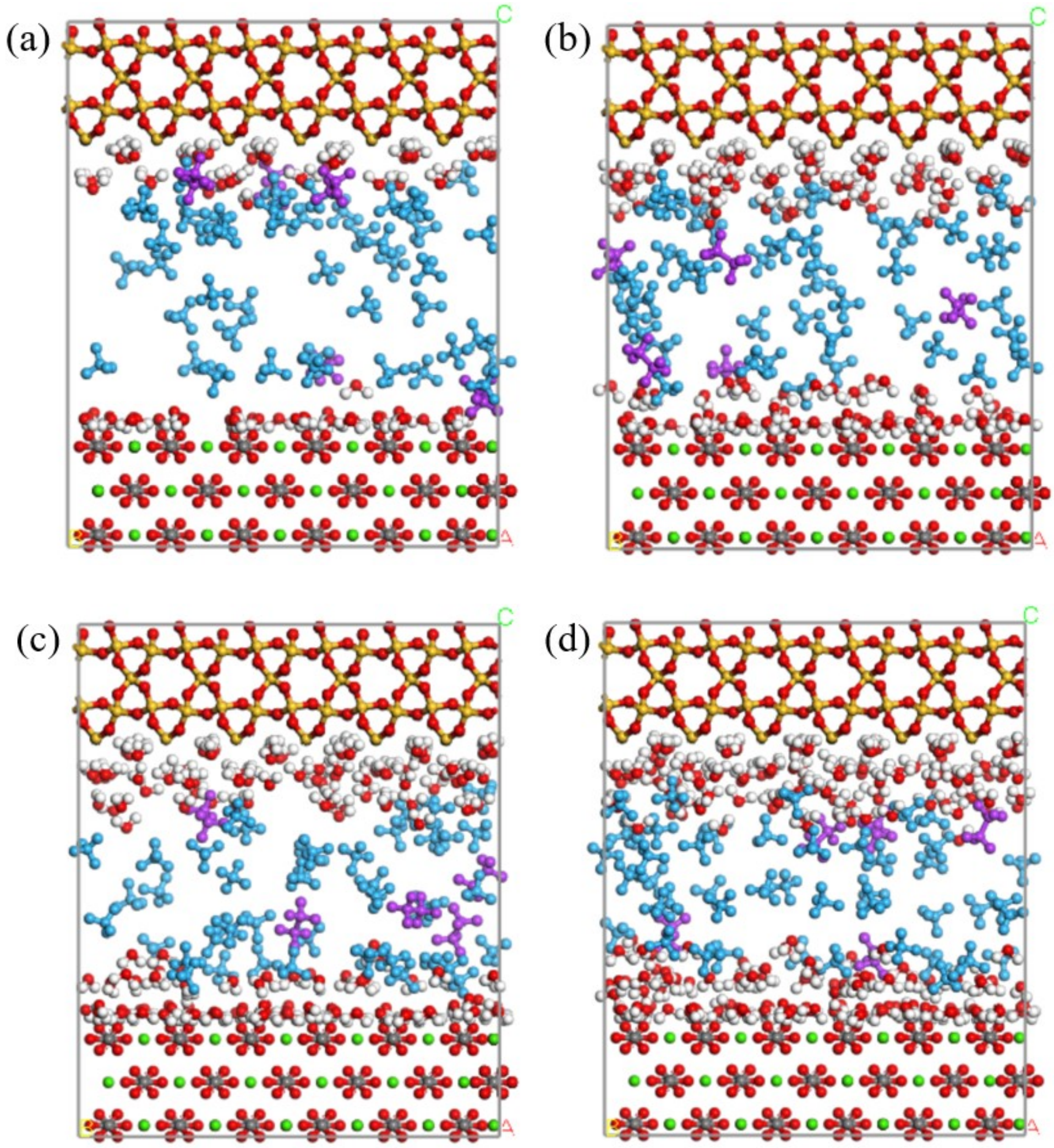

Fig. S2. Initial models of quartz-calcite nanopores containing shale gas with different water content conditions, with water content of (a) 6 wt%, (b) 10 wt%, (c) 12 wt%, and (d) 15 wt% (blue represents CH_4, and purple represents C_2H_6).

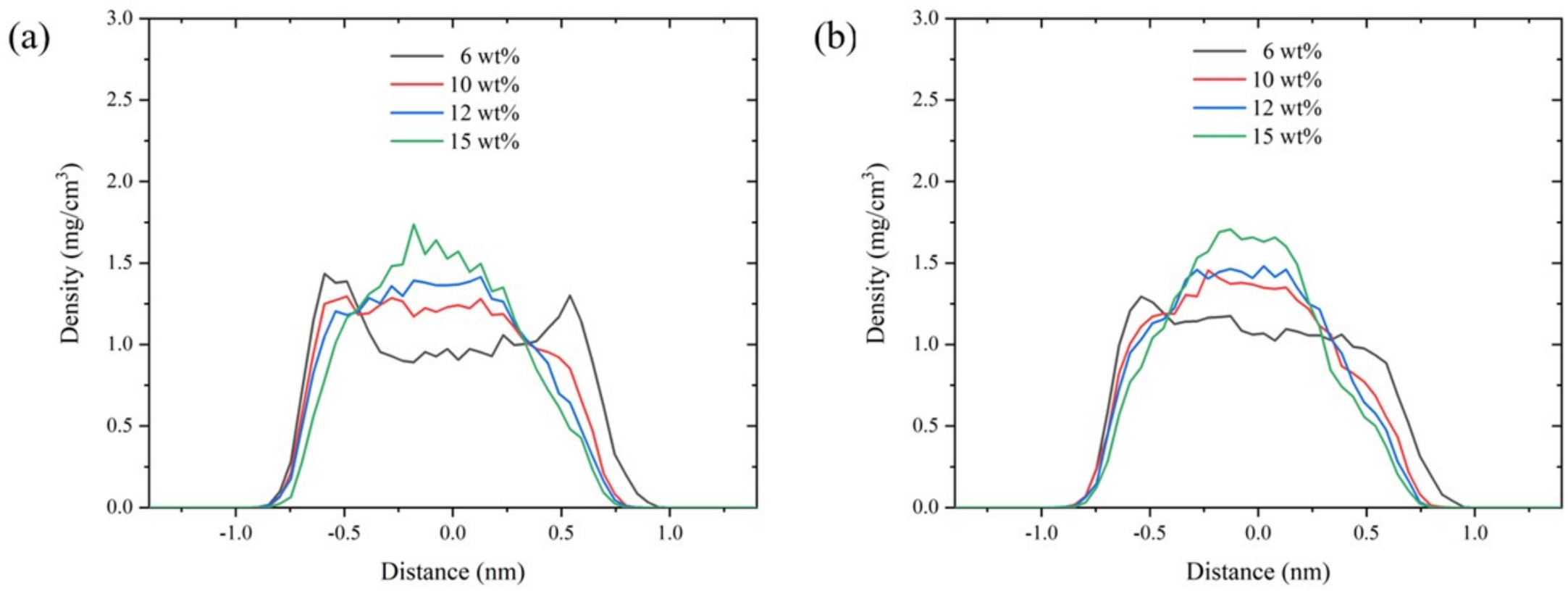

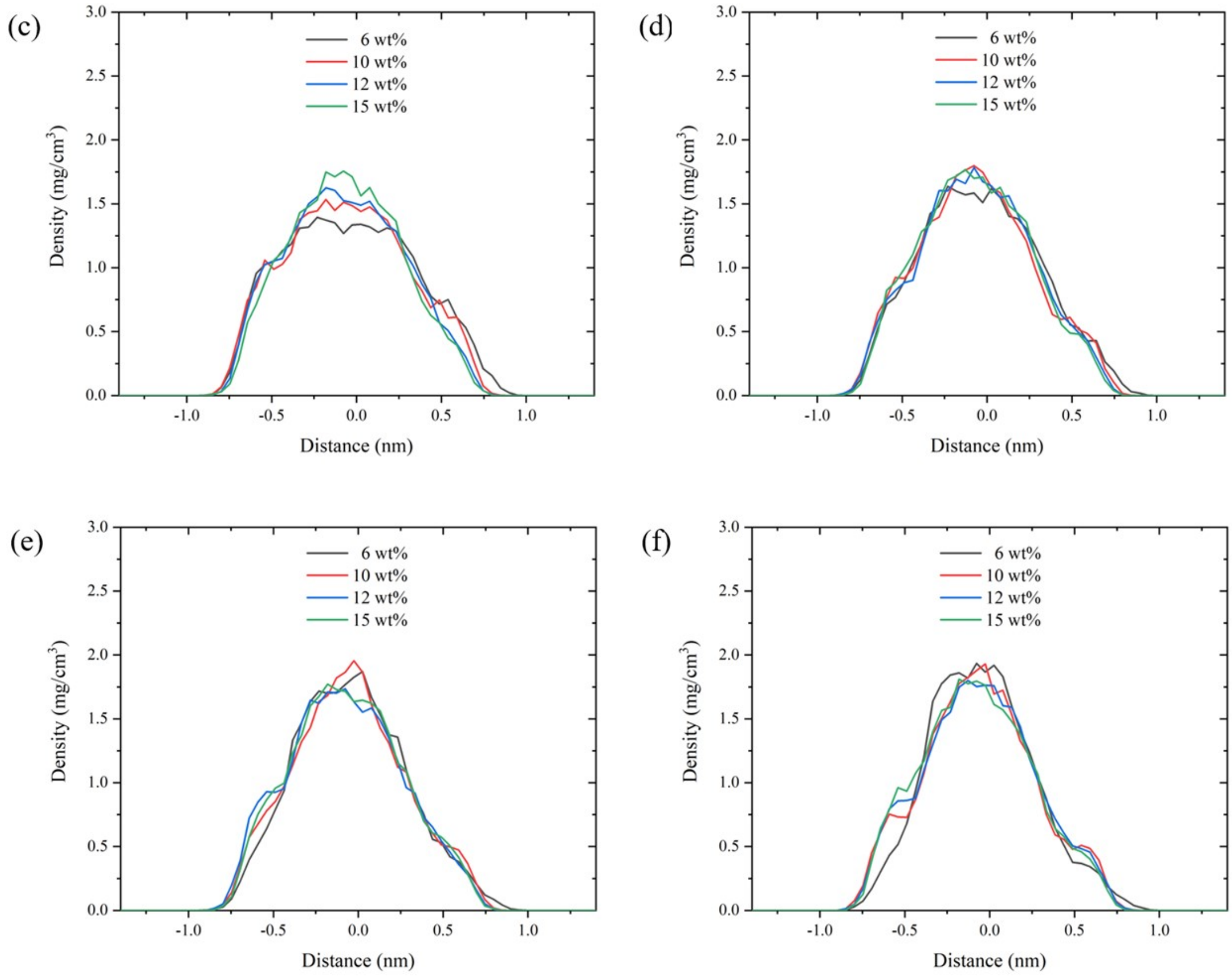

Fig. S3. Density profiles of CH_4 in water-containing and shale gas-containing quartz-calcite nanopores at 333.15 K and different pressures, with pressures of (a) 1, (b) 5, (c) 10, (d) 20, (e) 30 and (f) 60 MPa.

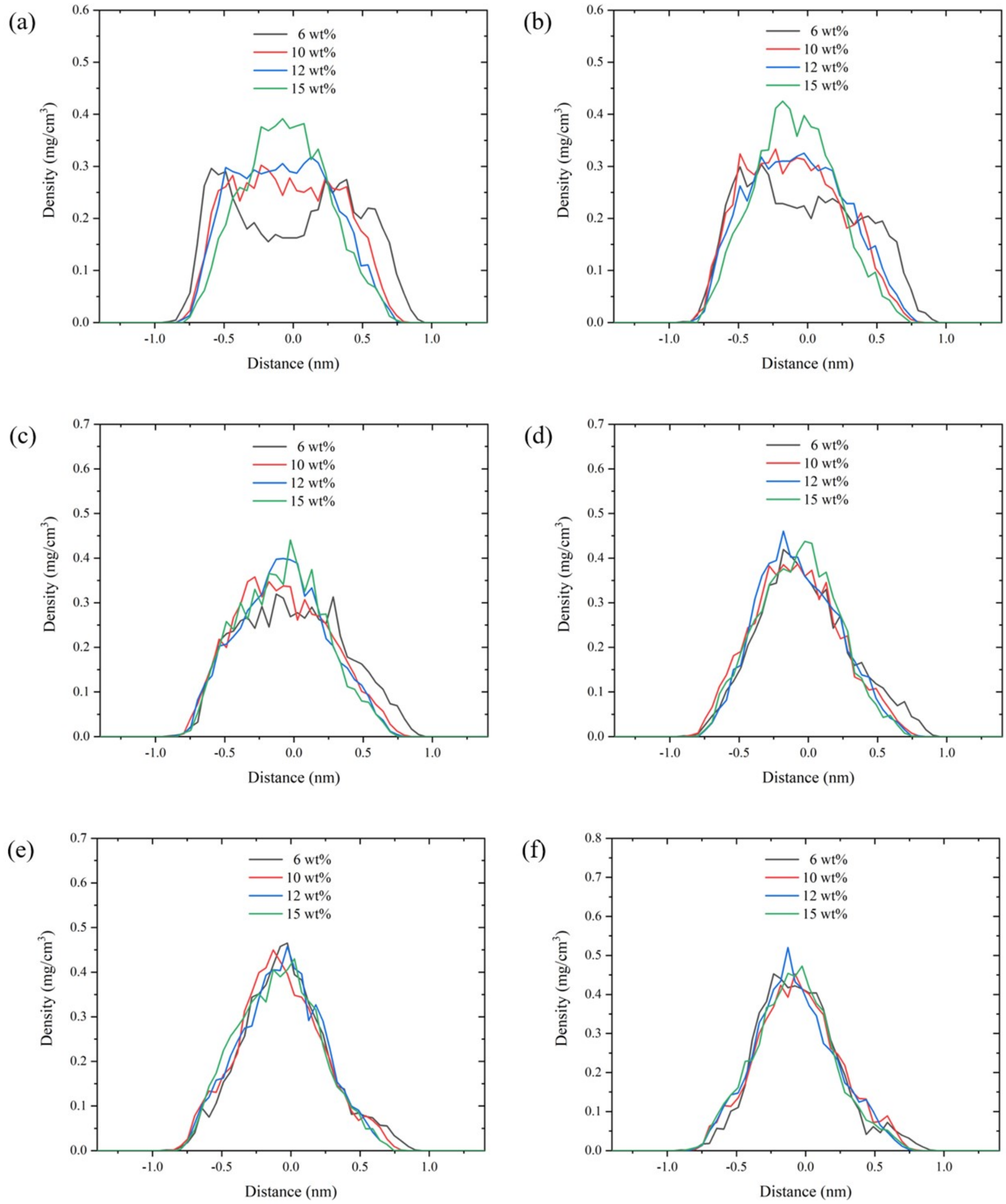

Fig. S4. Density profiles of C_2H_6 in water-containing and shale gas-containing quartz-calcite nanopores at 333.15 K and different pressures, with pressures of (a) 1, (b) 5, (c) 10, (d) 20, (e) 30 and (f) 60 MPa.
